# P03-010-B - A novel mutation in MEFV gene is not enough

**DOI:** 10.1186/1546-0096-11-S1-A206

**Published:** 2013-11-08

**Authors:** D Martorana, M Carrabba, G Fabio

**Affiliations:** 1 Unit of Molecular Genetics, University Hospital of Parma, Parma, Italy; 2 Internal Medicine Department and Medical Science and Community Health, Fondazione IRCCS Ca' Granda, Italy; 3 Universita' degli Studi, Milan, Italy

## Introduction

Genotype-phenotype correlation is still challenging in FMF patients especially when the disease is part of a complex autoinflammatory disease.

## Case report

A 23-year-old female was diagnosed for FMF in 2002 after a 3-year course of recurrent atypical attacks. Administration of colchicine 1mg was started and symptoms improved.

In 2005 she developed fever and epilepsy. CT-scans demonstrated splenic and cerebral lesions of uncertain etiology. Autoantibodies tests were negative. Infectious diseases were ruled out and therapy with steroids and carbamazepine was started under suspicious of vasculitis. Patient improved and in 2006 brain MRI showed resolution of cerebral lesions. She tapered steroids, gave up carbamazepine and was well under colchicine 1mg with rare mild FMF attacks but no signs of amyloidosis. She got pregnant in 2012. During pregnancy she experienced several mild FMF attacks with slight improvement despite colchicine increasing to 1.5mg. She developed gestosis. Her delivery was complicated by big vaginal hematoma that was surgically drained in two subsequent times. Further investigations on haemophilic acquired diseases were negative as well as platelets functional tests.

DNA sequence analysis did not find classic MEFV mutations and revealed heterozygosity for a novel c.460T>C in exon 2 , leading to the replacement of a polar Serine by an hydrophobic Proline at amino acid position 154 (S154P), not reported in Infevers database. This nucleotide substitution was not present in the genomic DNA of 734 control chromosomes. No mutations of the exons 2 and 11 of the Mevalonate Kinase gene and the exons 2,3,4 and 6 of the TNFRSF1A gene were found The woman was found to be heterozygous for the c.362G>A in the exon 4 (R92Q). Her father is heterozygous for the R92Q low penetrance variant in the TNFRSF1A gene, while her mother is heterozygous for the S154P mutation in the MEFV gene; both the parents do not report any inflammatory attack and are asymptomatic for autoinflammatory syndromes.

## Discussion

About 200 MEFV gene mutations have been described so far in patients with FMF. Recently it has been shown that in up to 25% of the patients, one MEFV mutation is probably enough to manifest the disease. This behavior could be explained assuming an interaction between the single MEFV mutation with another mutated inflammatory gene. R92Q is considered a low penetrance TNFRSF1A mutation that has been found to have a population frequency between 2-5%. The role of the R92Q has not been wholly understood. Given the fact that the carrier rates for R92Q are very similar and do not significantly differ from the carrier rate in controls, it might be concluded that an interaction between TNFRSF1A and MEFV is minimal or does not exist. A number of patients with heterozygous mutations in two autoinflammatory genes have been described so far in MEFV-TNFRSF1A, MVK-TNFRSF1A, and CIAS1-MEFV. The scarcity of such patients provides support to the conclusion that these autoinflammatory genes do not interact.

Based on the notion that some individuals who harbour only one mutation may have the second hit in currently unrecognized autoinflammatory genes, a whole-exome sequencing in selected cases may be performed.

## Competing interests

None Declared.
